# The Change in the Journal

**Published:** 1892-04

**Authors:** 


### *EDITORIAL.*

Address communications relating to the Editorial Department to DR. L. B. GRANDY, Atanta, Ga., Box 431.

Business communications should be addressed to DR. M. B. HUTCHINS, Atlanta, Ga.

All remittances should be made payable to "ATLANTA MEDICAL AND SURGICAL JOURNAL."

Articles for publication should reach this office not later than the 15th instant.

The editors are not responsible for views expressed by contributors.

### THE CHANGE IN THE JOURNAL.

The continued illness of Dr. L. P. Kennedy has induced him to dispose of his interest in THE JOURNAL to his associates, Dr. Hutchins and Dr. Grandy. We regret the unfortunate circumstances that have brought about this change, and trust that Dr. Kennedy's sojourn at his former home in South Carolina will fully restore his health. In parting with the assistance of Dr. Kennedy his colleagues cannot pay too high a tribute to him, both personally and in a business way; a gentleman of the highest character, always honest and absolutely reliable.

For some time certain changes in the typographical appearance of THE JOURNAL have been contemplated, and the present juncture has seemed to us a favorable occasion for them. Our cover and title page have been changed, heavier paper has been substituted at some additional expense, and some minor alterations have been made, all of which combine, we think, to make a decided improvement in our appearance.

It will not be the policy of this JOURNAL to stand still. Whenever or wherever any alteration may be made which can work for the interests of THE JOURNAL's patrons it will be promptly done. We expect to give our time and labor to this publication, and, with the aid of our able and deservedly well known staff of co-operators, we shall endeavor to establish a medical journal in Atlanta which shall have no superior in the South. To this end, also, we invite the kind assistance of our subscribers everywhere, from whom we hope to receive at any time original communications, therapeutic notes, or even small news of medical interest.

Nowadays it is almost a weekly occurrence for a new medical journal to be conceived and brought forth. Some appear healthy, exhibit great lung power, and promise well at first, but gradually perish from inanition. This JOURNAL is no experiment. It is now in the forty-seventh year of its publication, and there was never a time when it was on so sure and substantial a basis as it is to-day. Under our administration we do not fear that THE JOURNAL will take any steps backward.

We wish to state, in closing, that the delay in the appearance of THE JOURNAL this month has been unavoidable by reason of the changes made, and from the further fact that our publishers, Messrs. Harrison & Co., just at the time when we usually go to press, were moving their entire printing plant into their new building. We will try to reach our readers hereafter promptly on the first of the month.

